# Enhancing public health surveillance: A statistical validation of potential sampling bias in large retrospective vaccine cohorts

**DOI:** 10.3934/publichealth.2026031

**Published:** 2026-05-13

**Authors:** Marco Roccetti

**Affiliations:** Department of Computer Science and Engineering, University of Bologna, Bologna, Italy

**Keywords:** one health surveillance, global health, biostatistics, computational epidemiology, inferential statistics, public health data

## Abstract

In the context of Global Health, massive administrative datasets have become indispensable tools for health surveillance. However, the sheer scale of Big Data can mask systemic selection biases that standard mathematical adjustments may not fully mitigate. In this study, I propose a methodological audit of a recent large-scale cohort (N = 2,975,035) concerning COVID-19 vaccination and oncological outcomes. By benchmarking the cohort's architecture against national demographic and epidemiological gold standards through single-proportion Z-tests, we identified notable structural divergences. The first inferential test yielded a Z-score of −260.39 (p < 10⁻⁵⁰), suggesting a structural under-sampling of the elderly population (32.2% deficit) relative to the reference population. The second test identified a statistically inconsistent cancer incidence deficit in the non-vaccinated control group (Z = −15.23, p < 10⁻⁵⁰). These findings indicate that the reported statistical signals may emerge as a computational consequence of structural selection bias, where an artificially deflated baseline in the control group potentially inflates Hazard Ratios. Within a One Health approach, ensuring the structural integrity of data is crucial for effective prevention and control measures. We conclude that large-scale surveillance studies could be inferentially validated against demographic benchmarks to ensure that public health conclusions are grounded in baseline equivalence, thereby safeguarding the reliability of global health monitoring.

## 1. Introduction

The current landscape of Global Health surveillance is increasingly dependent on massive administrative health datasets, driving the adoption of complex epidemiological models. Within the One Health framework, this reliance is essential for monitoring disease trends; however, it introduces a significant challenge: The Big Data Paradox, where the vast scale of a dataset can mask systemic selection biases that standard mathematical modeling may struggle to fully mitigate. A core principle of biostatistics dictates that algorithmic complexity is subordinate to the structural integrity and external validity of the underlying data. We suggest that multivariable models, such as Cox proportional hazards regressions, may offer limited corrective power when comparison groups are fundamentally non-equivalent. In such cases, mathematical adjustment might not ensure sufficient control but instead potentially formalizes pre-existing statistical imbalances, leading to results that, while statistically significant, require careful epidemiological interpretation.

A large-scale retrospective cohort study, utilizing national administrative data, reported statistical associations suggesting an increased risk of cancer following COVID-19 vaccination [1]. Given the sensitivity of these issues for public health policies, those findings represent a methodological test case for data integrity. Our preliminary analysis identified a dual structural asymmetry by benchmarking the cohort in [1] against national demographic and epidemiological gold standards [2]–[7]. These observations established a methodological divergence that I seek to validate through formal inferential analysis. This divergence is based on two fundamental points. The first, demographic non-representativeness, is characterized by a systemic under-sampling of the elderly population (≥65 years). Since age is the primary predictor of oncological risk, this imbalance indicates a structural sampling divergence. The second, baseline risk deflation, is an observed 45.1% deficit in cancer incidence within the non-vaccinated high-risk control group (≥65 years) compared to national benchmarks [2]. This imbalance is the epidemiological consequence of the demographic asymmetry and serves as the mathematical prerequisite for the potential distortion of the study's primary outcome metrics.

My objective of this research is to statistically and inferentially validate these findings using rigorous Z-tests. Our central hypothesis is dual and causally linked: First, that the cohort's age distribution is statistically inconsistent with the source population; and second, that the deviation of the control group's cancer incidence from national rates is relevant to a level that rules out random chance. Inferential confirmation of this dual structural imbalance provides strong evidence that the reported signals emerge as statistical incongruences derived from uncorrected baseline non-equivalence.

Methodologically, it is also to be noticed that the techniques proposed in this study build upon and extend the structural framework recently established and successfully applied for detecting selection biases in observational cohort data [8],[9]. Specifically, this work advances the use of age-specific incidence rates analysis as a necessary tool to inform vaccine safety surveillance within a global health perspective, ensuring that age-specific incidence rates do not systematically deviate from gold standards.

The results we provide essentially confirm our hypotheses. The first Z-test (Z-score: −260.39) identified a profound sampling divergence (−32.2% deficit) due to the under-sampling of the high-risk elderly demographic (aged equal or over 65 years). The second Z-test (Z-score: −15.23) suggested a cancer incidence deficit (−45.1%) in the non-vaccinated control group, indicating the baseline risk was artificially deflated. Our study has hence formally validated a dual structural imbalance in a large COVID-19 vaccine cohort that previously reported increased cancer risk. In essence, this combined evidence suggests that the reported Hazard Ratios of the original study are highly likely to be statistical departures from gold standards, meaning the original study's findings should be interpreted with extreme caution before being used to inform public health decisions. The remainder of this paper is organized as follows: In the Materials and Methods Section, I detail the application of two single-proportion Z-tests used to inferentially evaluate the aforementioned representativeness and incidence. In the Results Section, I present the obtained test statistics, followed by a Discussion Section where I interpret the structural nature of the bias, its implications for the results' validity, and limitations. In the last section, the Conclusion Section, I formally address the methodological imbalance.

## 2. Materials and methods

### 2.1. Data sources, extracted metrics, and descriptive findings

The total number of participants in the final matched study cohort of [1] was 2,975,035 individuals. All other primary data, including Crude Incidence Rates (CRs), sample sizes (*N*), and age metrics, were extracted from that published paper and reported in Table 1. The national gold standard benchmarks were sourced from official South Korean cancer statistics [3]–[5] and demographic data [6],[7]. Based on these national figures, the population aged ≥65 years constituted 18% of the total population, and the crude incidence rate (CR) for this age bracket population was 155.2 per 10,000 individuals, as reflected in Table 1.

**Table 1.** Input parameters and national benchmarks for inferential *Z*-Tests.

Test	Parameter	Symbol	Value
Incidence (external validity)	National incidence (≥65 years)	*P(*0*)*	155.2 per 10,000 (0.01552)
	Observed incidence (≥65 years, Non–Vacc.)	*p(inc)*	85.2 per 10,000 (0.00852)
	Non-Vaccinated sample size (≥65)	*N(inc)*	72,285 participants
Age representativeness	National proportion (≥65)	*P(age)*	18.0% (0.18)
	Total cohort size	*N(Total)*	2,975,035 participants
	Total cohort *≥*65 proportion	*p(age)*	12.2% (0.122)

To this regard, it is worth reminding that, while socio-economic confounders undoubtedly influence cancer risk, age is prioritized as the primary audit metric due to its dominant role in oncological incidence [10]. In this context, the structural imbalance of the age distribution has served as a sufficient and necessary proxy for identifying systemic selection bias, regardless of other secondary variables. This prioritization enables a clear identification of first-order structural divergences that multivariable models might otherwise formalize rather than correct.

### 2.2. Inferential method I: Z-Test for the age representativeness

This test was used to formally evaluate the descriptive observation that the cohort's age composition was non-representative. The test compared the observed proportion of individuals ≥65 years in the cohort *p(age)*, that is *N(Cohort* ≥*65)* / *N(Total)*, against the established national proportion *P(age)*. The formal hypotheses could be consequently defined as follows:

Null Hypothesis (H0): The cohort's proportion of individuals aged ≥65 is not statistically lower than the national proportion: *p(age)* ≥ *P(age)*.

Alternative Hypothesis (H1): The cohort's proportion of individuals aged ≥65 is statistically lower than the national proportion (one-tailed test): *p(age)* < *P(age)*.

Based on well known epidemiological formulas [11], the *Z*-Score derivation can be calculated as in Eq 1:

$$Z\left(Age-Prop\right)=\frac{p\left(age\right)-P(age)}{\sqrt{\frac{P\left(age\right)\left(1-P\left(age\right)\right)}{N(Total)}}}$$ (1)

### 2.3. Inferential method II: Z-Test for the cancer incidence

This test formally verifies the hypothesis of a baseline cancer risk non-equivalence. It is carried out to have a confirmation/rejection of the hypothesis of consequence of the demographic imbalance on the cancer incidences. In essence, the test compares the observed cancer incidence rate in the non-vaccinated ≥65 subgroup *p(inc)* against the national gold standard rate *P(*0*)*. The formal hypothesis for this second test can be structured as follows:

Null Hypothesis (H0): The observed incidence rate in the cohort's ≥65 subgroup is not statistically lower than the national rate: *p(inc)* ≥ *P(*0*)*;

Alternative Hypothesis (H1): The observed incidence rate in the cohort's ≥65 subgroup is statistically lower than the national rate: *p(inc)* < *P(*0*)*.

Consequently, the *Z*-Score derivation can be formulated as follows in Eq 2:

$$Z\left(Incidence\right)=\frac{p\left(inc\right)-P(0)}{\sqrt{\frac{P(0)\left(1-P\left(0\right)\right)}{N(inc)}}},$$ (2)

In closing these Sections, first, it is to be noticed that the latter test and the former test were conducted as one-tailed tests, with a significance level of 5% to verify the hypothesized deficits. Second, we remind that the data presented here was either included directly or was extracted from the referenced documents. All calculations are easily reproducible based on the definitions provided. Further reasonable requests relative to data and calculations can be addressed to the corresponding and sole author of this manuscript.

#### 2.3.1. Ethics approval of research

This study constituted a methodological re-evaluation and secondary analysis of aggregated data published in peer-reviewed literature. There were no humans, animals, or plants involved, and no primary individual-level data were collected or accessed for this research; therefore, institutional review board approval was not required. The national epidemiological benchmarks used for comparison were derived from public reports accessible through official national health ministry repositories or through the referenced literature [1],[3]–[7]. I commit to providing the calculation spreadsheets and statistical code used for the inferential analysis upon reasonable request.

## 3. Results

The combined results of the two inferential Z-tests provide substantial statistical evidence of a dual structural imbalance, as detailed in the following two Sections.

### 3.1. Inferential analysis of demographic non-representativeness

The formal evaluation of the demographic deficit was obtained by comparing the observed proportion of ≥65 individuals in the cohort *p(age)* against the national benchmark *P(age)*. All calculations and results are provided in detail in Table 2.

**Table 2.** Calculation of *Z*-Score for age representativeness: *Z(Age-Prop)*.

Step	Description	Formula / Input values	Calculated value
I.A	Cohort proportion *p(age)*	*N(Cohort* ≥*65)* / *N(Total)* = 361,425 / 2,975,035	approx. 0.122
I.B	National proportion *P(age)*	National Benchmark	approx. 0.18
II	Numerator calculation	*p(age)*−*P(age)*	0.122−0.18 = −0.058
III	Standard error calculation	$\sqrt{\frac{P(age)\left(1-P\left(age\right)\right)}{N(Total)}}$	0.000223*
IV	*Z*-Score calculation	II / III	−0.058 / 0.000223 = −260.39

For display purposes in the Table, the value 0.000223 is approximated like the one in the column; the real value is 0.00022274..., which is the one used in the Z-score calculation. In the end, as seen from the final row in Table 2, the resulting test statistic is: *Z(Age−Prop)* = −260.39. The resultant p-value of <10⁻⁵⁰ leads to the rejection of the Null Hypothesis of demographic compatibility. This Z-score value formally establishes that the study cohort is structurally inconsistent with the source population, confirming the systemic relative deficit of −32.2% identified in the preliminary analysis of [2] for the highest-risk demographic.

### 3.2. Inferential analysis of cancer incidence divergence

The formal evaluation of the cancer incidence deficit (calculated as large as 45.1% in [2]) is obtained by comparing the observed rate in the non-vaccinated ≥65 subgroup *p(inc)* against the national gold standard, *P(*0*)*, using Eq 2, and yielding the results shown in Table 3.

As seen from the Table, the resulting test statistic is: −15.23. This *Z*-score is of an unprecedented magnitude, corresponding to a p-value that is statistically negligible (<10⁻⁵⁰). The Null Hypothesis is conclusively rejected, formally establishing that the −45.1% deficit in cancer incidence, identified in the preliminary analysis [2], is a structural divergence of risk.

In closing this Section, my inferential results establish a statistically evident chain of imbalance. The demographic non-representativeness of the cohort *Z(Age – Prop)* = −260.39, due to the under-sampling of the elderly, creates a baseline population that is structurally younger than the reference standard. Since older age is the primary risk factor for cancer, this structural issue leads potentially to the artificially deflated cancer incidence of 45.1% in the reference non-vaccinated group, thereby creating the prerequisite mathematical condition for the potential departure from standards of the results of [1].

**Table 3.** Calculation of the Z-score for cancer incidence: Inferential verification of baseline risk non-equivalence against the national standard.

Step	Description	Formula / Input values	Calculated value
I.A	Observed incidence *p(inc)*	85.2 per 10,000	85.2 / 10,000 = 0.00852
I.B	National incidence *P(*0*)*	155.2 per 10,000	155.2 / 10,000 = 0.01552
II	Numerator calculation	*p(inc)* – *P(*0*)*	0.00852−0.01552 = −0.00700
III	Standard error calculation	$\sqrt{\frac{P(0)\left(1-P\left(0\right)\right)}{N(inc)}}$	0.000460
IV	*Z*-score calculation	II / III	−15.23

## 4. Discussion

Because vaccine safety surveillance is a critical component of Global Health, it is essential to verify if reported signals are grounded in biological effects or emerge from structural features of the data collection process. I deemed it essential to audit the mathematical foundation of recent findings suggesting a link between COVID-19 vaccination and oncological risk. Hence, my goal of this research was to evaluate if the cohort proposed in [1] precisely represented the reference population, or if hidden statistical imbalances made the results look more alarming than they actually were. In this regard, it must be recognized that the utilization of big medical data for public health surveillance presents general inherent challenges; the sheer volume of administrative records can often create an illusion of precision where selection biases are not eliminated by the scale of the data, but rather masked or amplified. Addressing these complexities requires a continuous methodological dialogue to ensure that surveillance tools remain calibrated against real-world population benchmarks.

The inferential analysis of demographic divergence I conducted, established by a Z-score of −260.39, constitutes the fundamental finding of this audit. This value does not merely indicate a statistical difference; it represents a departure of the study cohort from the source population. The under-sampling of the high-risk elderly demographic (aged equal or over 65 years) at such a statistically improbable magnitude identifies a noteworthy sampling imbalance. Within the One Health surveillance perspective, this cause may render the use of the dataset non-representative and, consequently, limit the generalizability of any derived epidemiological conclusions.

The second inferential result (Z = −15.23) reflects a potential consequence of this demographic asymmetry. The value of this Z-score suggests that the 45.1% shortfall in cancer incidence in the non-vaccinated group could be interpreted as a statistical issue rather than a biological phenomenon. The statistical link is inferentially evaluated: By under-sampling the elderly, the researchers created a control group with a deflated baseline risk. This suppressed risk serves as a mathematically skewed denominator in the Hazard Ratio (HR) calculation. Being HR = Risk in Vaccinated Group /Risk in Non-Vaccinated Group (Deflated Baseline), an inflated HR may simply emerge as a computational consequence of the denominator's deflation given this mathematical architecture.

It is also worth mentioning that a potential technical concern in large-scale administrative data analysis is overdispersion or the violation of the independence assumption, which can artificially inflate Z-statistics. However, the extreme magnitude of the Z-scores I achieved in my audit (specifically Z = −260.39 and Z = −15.23) effectively insulates my conclusions from such artifacts. Even assuming a highly conservative variance inflation factor typical of clustered healthcare data, the null hypotheses would remain rejected, as the observed structural divergences far exceed any plausible threshold for random or systematic noise [12].

It is crucial to emphasize as a warning that I do not intend to deconstruct the biological findings per se, nor do I aim to draw definitive conclusions on the presence or absence of a causal link between COVID-19 vaccination and oncogenesis. Rather, I aimed at a global surveillance of cancer signals, providing a realistic methodological validation and demonstrating that the analyzed data structure does not confirm such associations due to baseline non-equivalence.

In essence, the Hazard Ratios reported in [1] may be viewed as mathematical echoes of pre-existing sampling imbalances. My dual evidence (Z = −260.39 and Z = −15.23) suggests that the multivariable models employed may have faced limited corrective capacity due to the structural asymmetry of the input data. This identifies a two-fold methodological challenge: i) A baseline inconsistency, where the outcome variable was skewed at the source; and ii) a computational inefficacy, where the scale of demographic non-representativeness exceeded the corrective capacity of standard statistical adjustment. This result finds a significant parallel in the literature [8],[9], where a similar framework was successfully applied to show how algorithmic complexity cannot substitute for initial data consistency in public health reporting.

A potential limitation of my analysis is the reliance on external national gold standards rather than individual patient-level data. While this comparative approach might appear strictly tied to the availability of national statistics, it actually represents a scalable logic of validation. The extreme magnitude of the obtained Z-scores robustly supports the validity of this method. In inferential statistics, Z-scores of this scale (exceeding −260) eliminate the possibility that unobserved confounders or minor regional deviations could account for the results. Indeed, the versatility of this framework can ensure that it is not restricted to national registries; it could be adapted to more limited research settings, such as multi-centric clinical studies, by substituting national benchmarks with validated local control populations or meta-analytical referents. My audit, in essence, has not only identified a marginal discrepancy in a specific case, but it can be a candidate for further applications across public health global surveillance initiatives [13].

The conclusion of this process is methodologically straightforward. By using rigorous statistical tests, I confirmed two major asymmetries: a) 32% fewer elderly people than the national average were included, making the group appear healthier than the real population; and b) as a consequence, the baseline cancer rate in the non-vaccinated group was nearly 50% lower than the gold standard. Therefore, the reported increase in cancer risk requires extreme caution in interpretation, as it appears to be a statistical departure from standards arising from the comparison of non-equivalent groups. Thus, my study serves as a constructive warning: In the era of Big Data, millions of records can lead to misleading conclusions if the data does not correctly represent the real-world population it aims to monitor [14]–[17].

## 5. Conclusions

Within the modern landscape of Global Health, the increasing integration of big medical data and automated modeling tools in epidemiology poses a critical challenge. If big-data-driven surveillance systems are applied massively without prior validation of their structural integrity, they risk formalizing and amplifying systemic selection biases, leading to misleading public health signals. In this era of computational epidemiology, ensuring baseline equivalence is not merely a statistical requirement but a fundamental safeguard for vaccine safety surveillance and public health policy.

In this context, my has provided robust inferential statistical evidence that the analyzed cohort represents an exemplar case of such risks, as it was characterized by a dual structural imbalance: A notable non-representativeness of the overall cohort relative to the source population (Z = −260.39) leading directly to a statistically inconsistent cancer incidence rate in the control group (Z = −15.23).

I conclude that the reported Hazard Ratios should be viewed as computational consequences resulting from an uncorrected structural asymmetry inherent in the cohort selection process. This research suggests that the validity of conclusions drawn from any large-scale cohort study must be conditional upon the inferential confirmation of methodological integrity against demographic and epidemiological gold standards. This inferential audit can be seen as not necessarily limited to national cohorts; it is a scalable methodology that can be adapted to multi-centric clinical studies by employing local validated sub-populations or meta-analytical benchmarks whenever national gold standards are unavailable. I close this paper by suggesting that, for effective Global Health surveillance, this inferential audit could become a standard prerequisite before any epidemiological findings are used to inform global health strategies.

## Use of AI tools declaration

The author declares that he has not used artificial intelligence (AI) tools in the creation of this article.
